# The Present and Future Status of the Dental Practitioner

**Published:** 1895-01

**Authors:** Jos. D. Hodgen

**Affiliations:** San Francisco, Cal.


### THE DENTAL REGISTER.

VOL. XLIX.] JANUARY, 1895. [No. 1.

### COMMUNICATIONS.

### The Present and Future Status of the Dental Practitioner.

Read in the Section on Dental and Oral Surgery, at the Forty-fifth Annual Meeting of the American Medical Association, held at San Francisco, June 5-8, 1894.

BY JOS. D. HODGEN, D.D.S., SAN FRANCISCO, CAL.

The dentist of to-day is the doctor of dental surgery, educated and licensed as such. As a practitioner his province embraces the science and art of diagnosing and treating diseased conditions of the organs and contiguous tissues of the oral cavity, operating surgically for deformed, injured and wasted conditions, and supplying artificial substitutes in the place of organs and tissues lost in that region.

From such it is to be inferred that the truly proficient practitioner should have, at least, a partial if not a finished medical education; that he is to no slight extent an oral surgeon, and that he is a skilful and scientific mechanic. He is thus at once a physician, a surgeon and a handicraftsman—a dentist. His parallel, to a partial extent, might appear in the oculist who linked with his own specialty the skill of the manufacturing optician.

In holding that a dentist should have the advantages of a medical education we are well aware that we meet with no inconsiderable opposition. But, while the opponents to such are strong in numbers, they are weak in point of argument to sustain their holding. It is argued that the time and expense necessary to such a course are its greatest hindrances. It is perhaps true that many men who are now enabled to pursue a course in dentistry would be deprived of that privilege if our colleges prolonged their term of pupilage to that extent necessary to embody a medical curriculum. However, we are led to doubt this as a fact, when we recognize that only three years ago the course in dentistry was lengthened from two years to three, and that by actual statistics there are more colleges and more students to-day than there has ever been at any time previous. Young men are crowding the colleges and the profession, who would do well to stay with the plough and farm. Are we, then, to lower the standard of the profession in order to receive these men to our already overflowing ranks? Is dentistry a refuge for those who are seeking what appears to them an easy and respectable means of gaining a respectable livelihood? It is also said that dental practitioners burdened with medical education "view their dental work through medical spectacles." What detriment this may be to their judgment as dentists is not clearly explained. Further, it is argued that the majority of dentists who attain a medical education "subsequently abandon dentistry for the practice of medicine." Such a condition does not interest us, or fall within our province to consider.

No; the status of the dentist of to-day is—change, preeminently above all else. He has reached that point at which he must declare himself a handicraftsman, a mechanic, a skilled laborer, or a scientific specialist of the great healing art. He has occupied the disputed position as long as progress will permit. Which shall it be? Back to mechanics and back to charlatanism, empiricism and closed laboratories, or a step towards medicine, a move towards occupying what has been termed the *terra incognita*, that territory of practice which has heretofore been partially claimed by both medicine and dentistry, and preempted by neither? It means that we must now decide whether we are to practice tooth restoration or tooth conservation; whether we are henceforth to be satisfied with the slaughter of natural organs and tissues, in order to restore and supply, or by scientific investigation aim at the conservation of what remains, and prevent the inroads of further destruction.

It has been lately remarked that "dentists are wonderful patchers-up of things half gone, but they do not show how to prevent the going." This is the key-note. And what is the cause of this condition? Is it because our mechanical skill has been neglected? Should we have had more infirmary and laboratory practice and training at college? Are our metallurgy and physics defective? No; more science and more medical education.

We feel safe in the assurance that conservative dentistry must necessarily be based on an appreciation of the anatomic, physiologic and pathologic relation of the mouth in disease and health, and that until the importance of this kind of knowledge is fully appreciated, a community will fail to receive at the hands of its dentists the benefit which they have a right to expect.

"To be impressed" says one, "with the necessity which these propositions indicate, one has only to remember the various important anatomic and physiologic functions of the mouth; its physical, organic and mental associations, and the corresponding extent of its pathologic relations.

"Anatomically, no portion of the human economy offers a more complex association of structures; physiologically, none whose functions are so diversified, and yet so essential; pathologically, none whose systemic relations are more significant.

"A consideration of the pathologic relations of the mouth includes radiated, reflex or sympathetic disturbances of harmony, or interference with function in contiguous or remote organs, originating in the mouth, as the expression in the oral cavity of lesions or interruption of harmony in other organs. Morbid systemic or constitutional conditions, whether the result of some intermediate primary affection, or of inherited tendency, or perversion, find usually a more or less strongly pronounced expression in the mouth.

"It is supposed that the dentist is entirely unacquainted with the systemic condition of his patient. Indeed, he is not presumed to have any right to meddle with such; while the physician rather complacently ignores the importance of the local lesions which is esteemed the province of the dentist to correct.

"Meanwhile, the patient chafes at the incompetency of the dentist who fails to arrest by mechanical skill the ravages of caries, and perhaps the operator himself fails to recognize in the lesions the expression of the systemic mischief. On the other hand, the physician, ignoring the local causes, tries in vain pills, and plasters, blisters, and purgatives; tries in vain the whole round of narcotics, sedatives, stimulants, tonics, anti-spasmodics, and anti-periodics; talking learnedly of neuroses and cachexia until pharmacopœia, patience and patients are alike exhausted."

These undeniable facts are illustrative in the necessity that a wider range of knowledge by the dentist is imperatively demanded.

During the year 1881 a considerable agitation of the subject, the status of the dentist; was evoked by the publication of a paper written by Dr. Truman W. Brophy, and read before the New York Odontological Society, in which that gentleman advocated the establishment of a Section of Dentistry in the AMERICAN MEDICAL ASSOCIATION. And notwithstanding the somewhat considerable opposition on the part of the physicians and dentists, that Section was organized at the thirty-second annual meeting of the ASSOCIATION. The principal objection to the movement at the time was, as many may remember, the condition upon which dentists were to become members. Since only those practitioners of dentistry who were graduates of regular medical colleges and members of local county societies were held eligible, the opposing members of the dental profession held that dentistry was being represented by representatives who had no constituency.

In the same year the first meeting of the Section on Diseases of the Teeth—a compromise in name—was held at the International Medical Congress in London.

At the thirty-eighth annual meeting of the AMERICAN MEDICAL ASSOCIATION, in 1887, to relieve the embarrassment that existed between the regular profession of medicine and the dental profession, it was argued that the Department of Dental and Oral Surgery was as much a part of the profession of medicine as ophthalmology or otology, or any other 'ology, "because," said our friend, "our teeth are parts of our system, and are used more than any other part," and that since dentistry has owned with humiliation the soil from which it sprang and has since steadily advanced presumably to a decent respectability, that it was only just and proper that the arms of the AMERICAN MEDICAL ASSOCIATION be more widely extended to receive the foster child.

At this meeting the line of demarcation was drawn as per the following resolution :

" *Resolved*, That the regular graduates of such dental and oral schools and colleges as require of their students a standard of preliminary or general education and a term of professional study equal to the best class of medical colleges in this country, and embrace in their curriculum all the fundamental branches of medicine, differing chiefly by substituting practical and clinical instruction in dental and oral medicine and surgery, instead of clinical medicine and surgery, be recognized as members of the regular profession of medicine, and eligible to membership in this ASSOCIATION on the same conditions and subject to the same regulations as other members."

This is as far as the recognition of dentistry as a medical specialty by medicine has gone. Since 1887 no particular advancement has been made, certainly, on the part of medicine. Thus far we have defined the position of dentistry and shown what has been done on the part of those most active in the advocacy of its being a specialty of medicine.

Medicine, in its broadest sense, is the science and theory of disease and of remedies, the art of preventing, curing or alleviating diseases and lesions of the human body, and its highest form to-day is found in its specialism ; in other words, the medical art breaks away at once from the unity of the theory of disease, and while there is but one body of pathologic doctrine for either sex, for every period of life, and for every region and part of the organism, the practical art divides itself into departments and sub-departments, such as surgery, obstetrics, dermatology, ophthalmology, otology, etc. Dentistry, or more properly at this juncture, odontology, being the science or art of preventing, curing, or alleviating the diseases of the teeth, which form an important part of the human body, is therefore a department of the healing art.

Such a deduction is but a natural one, and true in the confines of its premises. Why, then, are we still clamoring without the gates for admission? Because to the doctor of medicine this is not strictly true. He is not so liberal. Medicine to him is only medicine when the art of preventing, curing or alleviating diseases of the human body is practiced by medical graduates of medical colleges. When teeth are conserved from disease or extracted when useless, it is dentistry.

So the status of dentistry to-day, as far as our medical brothers are concerned, is not that of a specialty of medicine, principally because we are not graduates in medicine. Are they right? To those who may believe that they are not, it is comforting to feel that though their maternal acknowledgment is so dear and their association so keenly desired, they shall not be permitted to sit sole judges of our relationship.

Other influences, both external and internal, are doing much to drag us from this fireside of art and science. The first, we are led to believe, is the necessary amount of mechanics our profession is obliged to teach and practice; 2, the distinctive degree and curriculum we are attempting to maintain and enforce; 3, the percentage of our profession who are not graduates—many of whom, though, are our brightest lights; 4, our determination to be recognized.

The forces and influences which are tending to draw us beneath the maternal roof are, however, such that they will eventually overcome all opposing ones. They are:

1. The addition of medical chairs to dental faculties, and the division of dentistry into the operative and the mechanical dentist.

In all large communities men who make a business of doing mechanical work for one or more practitioners are fast becoming a special department of dentistry. These mechanical dentists in many cities do a very large percentage of all the mechanical work, and in some States the law is inclined to wink at the infractions of these unlicensed.

2. The patent dissatisfaction in which the degree is held, and the disinclination of colleges and universities to settle upon the not unusually adopted degree of doctor of dental surgery, together with the tendency to establish a variety of post-graduate degrees for the purpose of advertising those decorated and the college conferring it.

3. The rapid percentage decrease of the body of non-graduates and the tendency of dental and medical graduates who intend to practice dentistry to acquire both medical and dental degrees.

4. The establishment of the Oral Section of the AMERICAN MEDICAL ASSOCIATION.

The elevation of any particular class of men is, however, dependent upon a demand on the part of their constituents: the people. The greatest advancement of any profession or body: the pulpit, the bar, the medical fraternity, or what not, is the result of enlarged conception on the part of their representatives, and that conception has its stimulus in the public demand.

The forces, then, of successful issue lie in the education of our patients, in the establishment of recognition from them, occasioned by scientific efficiency, and an elevated qualification in the average practitioner.

No amount of recognition from a National, State or local medical association given a body of men undeserving, can be made to stand the test of popular opinion.

We therefore believe the future course of those who are so ably leading, to be not only through the channels of education as it may present itself in the education of dentists in the specialty of medicine, but that theirs is a plea for text-books and for education of the people.

Complete recognition can only, and will result, in the displacement of the dental degree by the medical for all, with special training for special practice.

### Local Anæsthesia.

A mixture of ten parts of chloroform, fifteen parts of ether and one part of menthol, used as a spray, is recommended (*Med. Age*) as an excellent and prompt means for obtaining local anæsthesia lasting for about five minutes.

